# MRI for breast cancer: Current indications

**DOI:** 10.4103/0971-3026.48431

**Published:** 2009-05

**Authors:** Haydee Ojeda-Fournier, Christopher E Comstock

**Affiliations:** University of California, San Diego, USA; 1 University of California, San Diego, Department of Radiology, Moores Cancer Center, La Jolla, California, USA

**Keywords:** Breast cancer, breast imaging, breast screening, mammography, magnetic resonance imaging, MRI, indications

## Abstract

Mammography is the only imaging study that has been proven in multiple large randomized trials to decrease breast cancer mortality. Mammography, however, has its limitations and, as such, other modalities that can complement it are being studied. One of these is dynamic contrast-enhanced breast MRI, which has emerged as an important adjunctive modality and is at present the most sensitive modality that we have to evaluate the breast. The American College of Radiology, in its 2004 practice guidelines, has outlined the 12 current indications for breast MRI. This manuscript reviews and provides examples of each of these.

## Introduction

Mammography is the only imaging study that has been shown in multiple large clinical trials to decrease mortality in breast cancer. However, mammography has well-known limitations; for example, it has limited use when there is increased breast tissue density, in the diagnosis of lobular carcinoma, in the postoperative breast, and in patients with BRCA and other gene mutations. As such, there has been an effort to develop other modalities to complement mammography. Dynamic contrast-enhanced breast MRI (DCE-MRI) has emerged as an invaluable adjunctive tool. The most recent American College of Radiology (ACR) practice guidelines for the performance of breast MRI[1] outline 12 indications [Table 1] for DCE-MRI. This article presents an example of each of these indications and reviews the literature in support of the recommendations.

**Table 1:** Current indications for breast MRI according to the American College of Radiology

Lesion characterization
Neoadjuvant chemotherapy
Infiltrating lobular carcinoma
Infiltrating ductal carcinoma
Axillary adenopathy, primary unknown
Postoperative tissue reconstruction
Silicone and non-silicone breast augmentation
Invasion deep to the fascia
Contralateral breast examination in patients with breast malignancy
Postlumpectomy for residual disease
Surveillance of high-risk patients
Recurrence of breast cancer

### Warnings and protocols

There is no standard recommended protocol for performing DCE-MRI. Protocols vary with the equipment being used and the clinician's preference. For example, while some clinicians favor evaluating images in the axial planes, others choose to interpret in the sagittal plane. There are, however, minimum standards for the performance of breast MRI and these are outlined in Table 2. A dedicated breast coil, at least 1.5-Tesla magnet strength, and dynamic contrast administration are absolute requirements for the performance of breast MRI. A power injector is highly recommended to standardize contrast administration from study to study.

**Table 2:** Minimum standards required for performing breast MRI

Field strength	Minimum 1.5-T
Resolution	3 mm slice thickness
Contrast	Gadolinium, 0.1 mmol/kg
Scan time	Dynamic contrast enhancement
Coil	Dedicated breast

Breast MRI should not be used instead of mammography; it is a complementary study to other breast imaging modalities. It is recommended that a current mammogram be available for comparison when interpreting the breast MRI. Finally, DCE-MRI should not be used in lieu of a biopsy of a suspicious lesion found by USG, mammogram, or physical examination, or for the evaluation of characteristically benign lesions. Women undergoing breast MRI should be advised that although the study is highly sensitive, its low specificity could lead to recommendations for additional imaging follow-up studies or biopsy.

A final but important warning is the need for the establishment of MRI-guided needle localization or biopsy capability in any breast MRI practice. Since there will be lesions found at the time of DCE-MRI that are clinically, mammographically, and sonographically occult, there needs to be a program in place to address such lesions.

### Current ACR practice guidelines

#### Lesion characterization

**Figure 1 (A,B):**
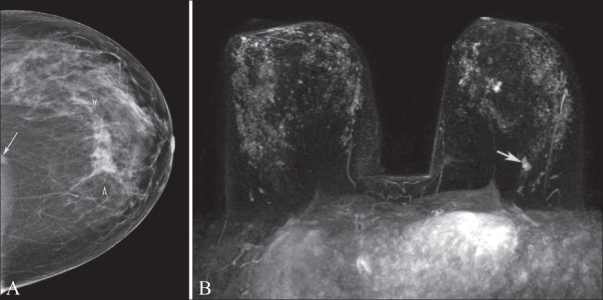
Mammogram, craniocaudal projection (A) and maximum-intensity projection (MIP) DCE-MRI (B). This was a 48-year-old woman noted to have a density (arrow) in the craniocaudal projection only and architectural distortion (arrowheads) in the subareolar location in both standard mammographic views. The anterior lesion was biopsied and proved to be invasive mammary carcinoma with ductal and lobular features. The density could not be identified on multiple additional diagnostic views. DCE-MRI was recommended to further evaluate the initial mammographic finding. The lesion (arrow) was identified by MRI and located at the 6 o'clock position, far posterior to the chest wall. This lesion was also biopsied and proven to be a second area of invasive mammary carcinoma

Lesion characterization is probably the weakest and least investigated indication for DCE-MRI.[2] However, when mammography and USG fail to fully evaluate a finding, we have found breast MRI to be a useful complementary study to conventional breast imaging modalities [Figure 1]. Although DCE-MRI is highly sensitive, the specificity and negative predictive value (reported, for example, by Bluemke *et al*,[3] to be 67.4% and 85.4%, respectively) are not sufficiently high to preclude biopsy when there are suspicious imaging findings.

#### Response to neoadjuvant chemotherapy

**Figure 2 (A,B):**
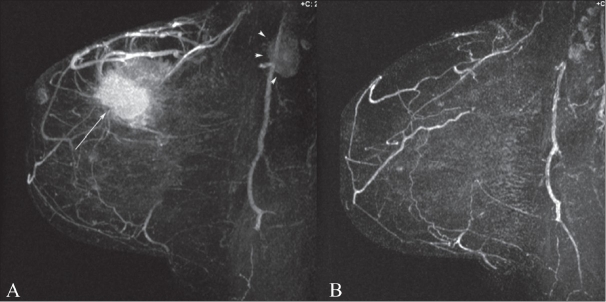
MIP DCE-MRI image before (A) and after chemotherapy (B). This was a 53-year-old woman with invasive ductal carcinoma, grade 3. Note the primary tumor (arrow) as well as the axillary lymphadenopathy (arrowhead). Complete response to chemotherapy of the primary tumor as well as complete resolution of axillary lymphadenopathy is noted after chemotherapy (B). At histopathology, there was no residual disease at the lumpectomy site and all axillary lymph nodes were negative

Neoadjuvant chemotherapy is routinely used in advanced breast cancers to reduce the size of the tumor so that conservation surgical therapy can be performed. MRI has been shown to be better than physical examination, mammography, and USG for assessing residual disease after neoadjuvant chemotherapy.[4,5] There are, however, limitations to DCE-MRI evaluation of residual disease after neoadjuvant chemotherapy. MRI tends to overestimate the size of residual disease and, because of the antiangiogenic effects of certain chemotherapeutic agents on tumor, the ability of DCE-MRI to evaluate lesion enhancement can be significantly decreased. Figure 2 demonstrates the pre- and post-chemotherapy MRI appearance of breast cancer in a patient who had complete response to therapy.

#### Extent of infiltrating lobular carcinoma

**Figure 3 (A,B):**
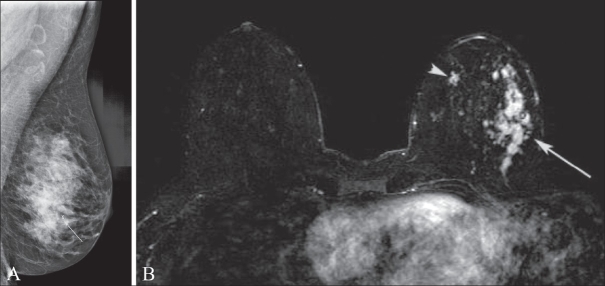
Mediolateral oblique projection mammogram (A) and fat-saturated axial T1W postcontrast (B) images. In this 60-year-old woman who desired breast conservation, DCE-MRI not only demonstrated extensive disease laterally (arrow in B) at the site of the mammographic finding (arrow in A) but also in the medial aspect of the breast (arrowhead in B). The medial breast was biopsied under MRI guidance and there proved to be an additional focus of lobular carcinoma. The surgical management in this case was mastectomy with breast reconstruction

Infiltrating lobular carcinoma is known to be a diagnostic challenge in mammography. It is often seen in only one projection and is well known to be underestimated by both mammography and USG.[6] Breast MRI has been shown in several studies to better depict the extent of lobular carcinoma.[7,8] In Figure 3 it can be seen that the DCE-MRI of a patient with heterogeneous breast tissue and known lobular carcinoma better depicts the extent of disease as compared to the mammogram.

#### Extent of infiltrating ductal carcinoma

**Figure 4 (A,B):**
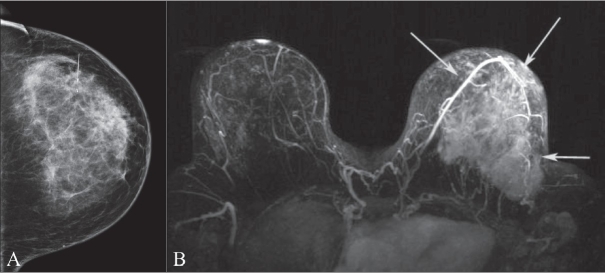
Craniocaudal mammographic projection (A) and MIP DCE-MRI image (B). This 42-year-old female was referred for DCE-MRI to determine the extent of disease after a new diagnosis of invasive ductal carcinoma. Note how much better MRI demonstrates disease (arrows in B) than the mammogram (arrow in A)

DCE-MRI has been shown in multiple studies to be capable of accurately evaluating the extent of infiltrating ductal carcinoma and finding additional, mammographically occult, areas of disease.[9–11] Evaluating the extent of disease in patients with newly diagnosed breast cancer is the most common indication for DCE-MRI at our institution. Figure 4 demonstrates the dramatic extent of disease revealed by DCE-MRI in a patient with recently diagnosed breast cancer. In this patient who clinically had inflammatory changes, the study also served to establish a baseline prior to neoadjuvant chemotherapy.

#### Axillary node metastases with an unknown primary

**Figure 5 (A,B):**
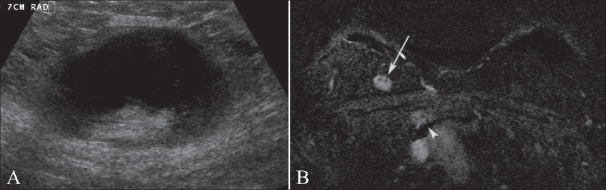
USG of an enlarged axillary lymph node (A) and fat-saturated axial DCE-MRI subtracted image (B). This was a 48-year-old woman who initially presented with a palpable lump in the axilla. Axillary metastasis (A) due to an unknown primary was confirmed. Breast MRI identified a suspicious lesion (arrow in B) in the upper inner quadrant of the right breast. A second-look USG and biopsy followed, which confirmed the lesion to be the site of a primary breast malignancy. Also note the internal mammary lymphadenopathy (arrowhead in B)

Occult breast cancer is an uncommon presentation of breast carcinoma. When axillary lymph node metastasis is identified and the mammogram is negative, breast MRI is able to locate the primary site in 75–86% of women.[12,13] Although identifying the primary breast tumor will not affect the prognosis in such cases, it will allow the patient to consider breast conservation surgery as a treatment option. Figure 5 shows a patient with biopsy-proven lymph node metastasis of a breast primary and a negative mammogram who had a positive breast MRI. Second-look USG was then performed and the lesion was identified and targeted for biopsy.

#### Postoperative tissue reconstruction

**Figure 6:**
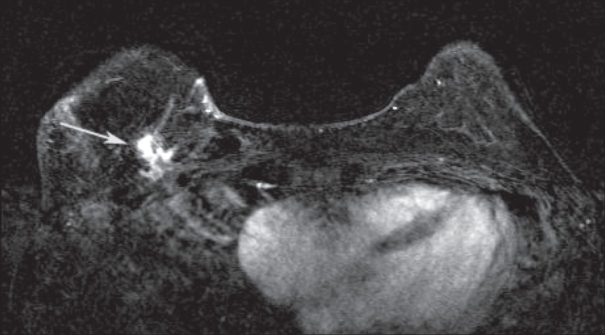
Axial subtracted DCE-MRI image of a patient with right TRAM flap reconstruction after mastectomy. This patient presented with a new thickening situated medially in the reconstructed right breast. DCE-MRI demonstrated a spiculated mass (arrow) with abnormal enhancement. The patient turned out to have widely metastatic disease at the time. She refused further evaluation or treatment

Many patients who have undergone mastectomy choose to have breast reconstruction with autologous tissue such as a transverse rectus abdominis myocutaneous flap (TRAM), latissimus dorsi flap, or gluteal flap. Discussion of the reconstructive technique is beyond the scope of this manuscript; briefly, the chest wall is covered with fatty tissue and in some cases an implant is added to give bulk to the reconstructed mound. Follow-up of these patients, both clinically and with mammography, gives only limited information. Mammography has not conclusively been shown to help in detecting recurrence,[14] but it can be used as part of routine surveillance in patients with a history of breast cancer. DCE-MRI, which can clearly depict the chest wall, is helpful in identifying local recurrent disease.[15] In addition, DCE-MRI can identify benign changes that can present clinical dilemmas in patients with autologous tissue reconstruction. Figure 6 demonstrates the case of a patient who presented with a new palpable abnormality at the site of a TRAM flap site and was noted to have suspicious enhancement. In this case, however, there was no histologic confirmation since the patient refused further evaluation and therapy.

#### Silicone and non-silicone breast augmentation

**Figure 7 (A–C):**
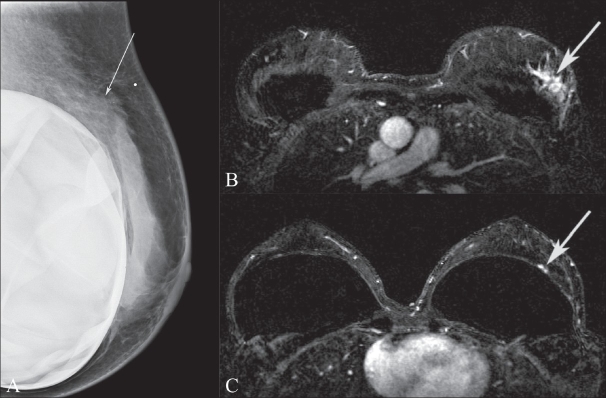
A 45-year-old female with a new palpable abnormality that was proven to be invasive ductal carcinoma. A mediolateral oblique mammographic projection of the left breast (A) with a BB in place at the site of the palpable abnormality demonstrates increased density (arrow) with associated calcifications. Preoperative DCE-MRI was performed (B,C), which revealed the breast carcinoma (arrow in B) as well as an additional focus of disease at the 3 o'clock position (arrow in C) which was not seen on mammography but was identified on second-look USG

Before the availability of gadolinium contrast enhancement, evaluation of implant integrity in patients with silicone breast augmentation was the first indication for MRI imaging of the breast. The many types of implants and the appearance of rupture have been nicely reviewed by Middleton and McNamara[16] In patients with implants, the mammogram may be difficult to interpret, and evaluation for the presence of cancer in such cases is another indication for DCE-MRI. An additional advantage of imaging with MRI is that it can visualize lesions behind the implant and it is advocated as the study of choice for evaluation of breast cancer in patients with implants.[2] Figure 7 demonstrates the usefulness of DCE-MRI in not only defining the extent of disease but also in finding additional foci of carcinoma in a patient with a breast implant. At our institution, for patients with silicone implants being evaluated by DCE-MRI, we perform implant-specific sequences. This procedure adds just minutes to the examination and provides useful information which, in the case of implant rupture, can be of clinical significance.

#### Invasion deep to fascia

**Figure 8 A–C):**
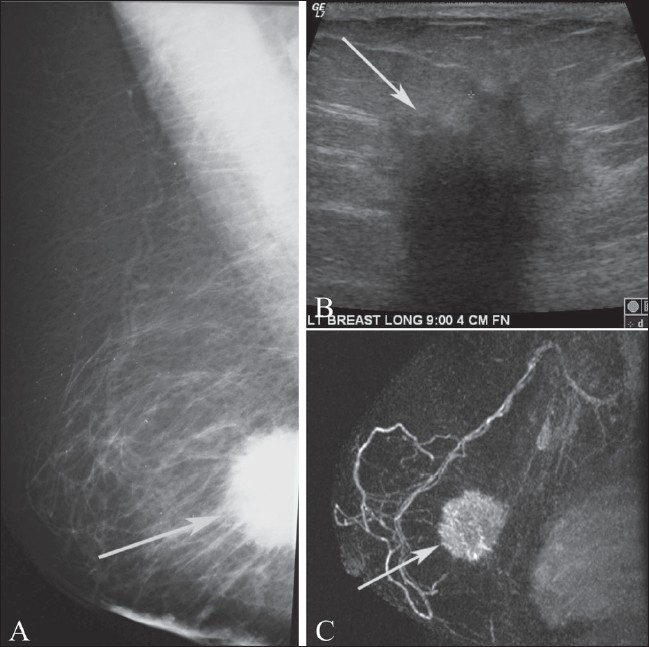
(Mediolateral oblique mammographic view (A) of a patient who complained of shrinking breast size. A spiculated mass (arrow) is seen in the posterior one-third of the breast, which was incompletely evaluated by mammography. The USG (B) demonstrates an angular and spiculated lesion (arrow) with posterior acoustic shadowing, which hinders evaluation of the fascia and muscle. Only the MIP MRI image (C) clearly demonstrates that although the retroglandular fat is obliterated, there is no chest wall invasion (arrow)

Both mammography and USG have limitations in the evaluation of the chest wall. MRI is able to visualize the entirety of the chest wall. Enhancement of the pectoralis and intercostal muscles is indicative of chest wall invasion in patients with a posterior breast tumor.[17] Obliteration of the overlying fat plane, in contrast, is not sufficient to suggest chest wall invasion. Knowledge of chest wall invasion is invaluable for preoperative planning. In Figure 8, the mammogram [Figure 8A] does not completely image the posterior chest wall. USG [Figure 8C], because of posterior shadowing caused by the tumor, gives no information on the status of the chest wall. The DCE-MRI [Figure 8C], in contrast, demonstrates no abnormal enhancement of the muscle, and at surgery there was no facial invasion.

#### Contralateral breast screening

**Figure 9:**
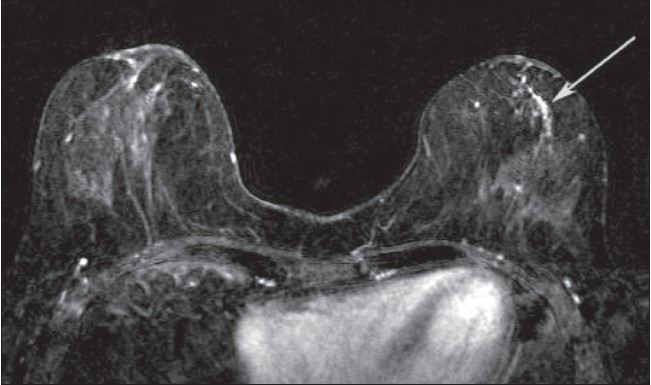
Axial DCE-MRI subtracted image in a patient with a known right breast cancer (not seen at this level) and unsuspected abnormal left breast ductal enhancement (arrow). The lesion on the left was proven to be ductal carcinoma in situe (DCIS) on MRI-guided breast biopsy

Women with a history of breast cancer are at increased risk for additional breast cancers. As many as 7% of women will be diagnosed with metachronous disease and up to 3% will have contralateral synchronous disease.[18] In recent studies, DCE-MRI has been able to identify occult contralateral cancer in 3–5% of the cases.[18,19] MRI, thus, is of value as a study to screen the contralateral breast in patients with a new diagnosis of breast cancer. At present, there is no data on the impact that DCE-MRI might have on survival when a synchronous contralateral tumor is identified. With the increased use of partial breast irradiation it will become even more critical to identify additional areas of disease. Figure 9 demonstrates the findings in a patient with a contralateral synchronous tumor. With high-resolution axial and parallel imaging it is now feasible to image both breasts simultaneously.

#### Residual disease post-lumpectomy

**Figure 10 (A,B):**
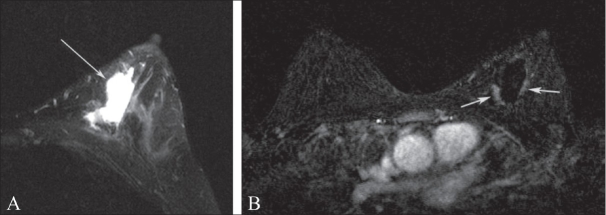
Axial T2W image (A) demonstrates a post-lumpectomy seroma cavity (arrow) in this patient with positive margins after conservation therapy for breast cancer. The axial DCE-MRI subtracted image demonstrates minimal thick, nodular enhancement in the posteromedial and lateral walls of the lumpectomy cavity (arrows). In this patient, re-excision of the cavity with attention to the areas noted on the DCE-MRI yielded negative margins

Lumpectomy followed by radiation is an acceptable choice in the treatment of stage I and II breast cancer and has been shown to provide the same survival as radical and modified radical mastectomies.[20] Positive margins are known to increase local recurrence rates. The rate of positive margins varies between surgeons but, in general, it is accepted that 40% of lumpectomies will have positive margins. The advantage of obtaining MRI prior to returning to the operating room for re-excision is that MRI helps in identifying multifocal or multicentric disease, which would change the management from lumpectomy to mastectomy.[21] MRI evaluation can also inform the surgeon as to the extent of residual disease and its location. Because of postoperative inflammatory changes, it is accepted that the specificity of DCE-MRI is limited in the postoperative period and that imaging earlier than 28 days post surgery will adversely affect accuracy.[22] Figure 10 shows how a patient with positive post-lumpectomy margins benefitted from DCE-MRI prior to re-excision.

#### Surveillance of high-risk patients

**Figure 11 (A,B):**
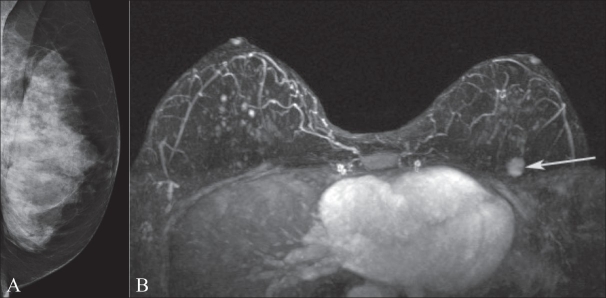
Mediolateral oblique projection of the left breast (A) in a BRCA1 gene mutation carrier. Note the extremely dense breast tissue. MIP postcontrast image (B) demonstrates an enhancing mass (arrow) in the upper left breast which is mammographically occult but was found at the time of second-look USG. Biopsy confirmed invasive ductal carcinoma

The generally accepted risk factors for breast cancer are outlined in Table 3. For patients with these risk factors there is sufficient evidence to recommend annual DCE-MRI in addition to annual mammography for screening for breast cancer.[23] In patients with genetic mutations, cancer is diagnosed at an earlier age, breast tissue is denser and, typically, the lesion is relatively larger in size at the time of diagnosis. There is insufficient evidence to recommend DCE-MRI screening in patients who have a personal history of breast cancer, prior atypical ductal hyperplasia, or other high-risk lesions at breast biopsy, and also in patients with heterogeneously dense or very dense breast glandularity. Figure 11 shows a case of mammographically occult breast cancer identified by DCE-MRI in a known breast cancer (BRCA1) gene mutation carrier.

**Table 3:** High-risk patients

BRCA1 or BRCA2 mutation, or untested first-degree relative of known carrier
Chest radiation between age 10–30 years for Hodgkin's lymphoma
Lifetime risk of 20–25% as determine by statistical risk assessment models such as BRCAPRO or Gail
Other genetic mutations, including p53 and Cowden

#### Recurrence of breast cancer

Evaluation of breast cancer in patients with autologous tissue or implant breast reconstruction has been described earlier in this manuscript. Postoperative changes are also known to hinder the evaluation of breast cancer recurrence at the lumpectomy site. Although there is little published data, MRI may be useful in evaluating for recurrent disease in patients in whom conventional imaging is confusing due to considerable postoperative scarring. While scar may enhance on MRI for 1–2 years following surgery, a negative MRI may be helpful in excluding recurrent disease. This may be more difficult when the postoperative scar is still enhancing. In general, a scar tends to present as a thin rim or cloud of enhancement around the cavity, whereas recurrent tumor tends to be more clumpy or mass-like.

**Figure 12 (A,B):**
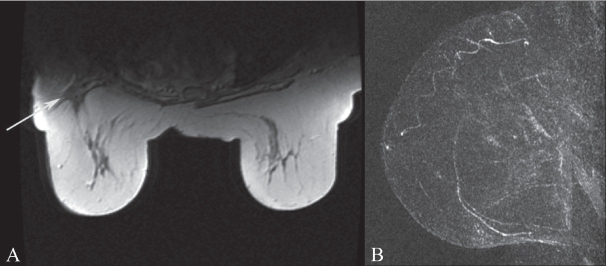
T1W non-fat-saturated axial image (A) demonstrates a scar in the right breast (arrow) in a patient with prior lumpectomy and questionable increasing architectural distortion on mammogram. The MIP image (B) of the breast demonstrates no enhancement of the scar, providing reassurance that there is no recurrence

## Conclusion

In this article we review the indications for DCE-MRI examination as per the current ACR guidelines and present examples for each of these indications. Breast MRI has emerged as the most sensitive modality for evaluation of the breast; however, it is limited by low specificity. Breast MRI does not replace mammography for screening of breast cancer in the general population. MRI-guided localization or a biopsy system and the requisite expertise are needed for any breast MRI program as there will be lesions that will not be seen by other imaging modalities.
